# Challenges Faced with Small Molecular Modulators of Potassium Current Channel Isoform Kv1.5

**DOI:** 10.3390/biom10010010

**Published:** 2019-12-19

**Authors:** Zefeng Zhao, Songsong Ruan, Xiaoming Ma, Qian Feng, Zhuosong Xie, Zhuang Nie, Peinan Fan, Mingcheng Qian, Xirui He, Shaoping Wu, Yongmin Zhang, Xiaohui Zheng

**Affiliations:** 1 Key Laboratory of Resource Biology and Biotechnology in Western China, Ministry of Education, Northwest University, 229 Taibai Road, Xi’an 710069, China; zzf598155752@sina.com (Z.Z.); ruansongsong@stumail.nwu.edu.cn (S.R.); 201720920@stumail.nwu.edu.cn (X.M.); fengqian@stumail.nwu.edu.cn (Q.F.); 18821657783@163.com (Z.X.); nz19980217@163.com (Z.N.); f568902@163.com (P.F.); yongmin.zhang@upmc.fr (Y.Z.); zhengxh@nwu.edu.cn (X.Z.); 2 Biomedicine Key Laboratory of Shaanxi Province, School of Pharmacy, Northwest University, 229 Taibai Road, Xi’an 710069, China; 3 Department of Medicinal Chemistry, School of Pharmaceutical Engineering and Life Science, Changzhou University, Changzhou 213164, China; mqian2019@cczu.edu.cn; 4 Laboratory for Medicinal Chemistry, Ghent University, Ottergemsesteenweg 460, B-9000 Ghent, Belgium; 5 Department of Bioengineering, Zhuhai Campus of Zunyi Medical University, Zhuhai 519041, China; xiruihe@163.com; 6 Sorbonne Université, Institut Parisien de Chimie Moléculaire, CNRS UMR 8232, 4 place Jussieu, 75005 Paris, France

**Keywords:** potassium channel, Kv1.5, KCNA5, modulators, SAR

## Abstract

The voltage-gated potassium channel Kv1.5, which mediates the cardiac ultra-rapid delayed-rectifier (*I*_Kur_) current in human cells, has a crucial role in atrial fibrillation. Therefore, the design of selective Kv1.5 modulators is essential for the treatment of pathophysiological conditions involving Kv1.5 activity. This review summarizes the progress of molecular structures and the functionality of different types of Kv1.5 modulators, with a focus on clinical cardiovascular drugs and a number of active natural products, through a summarization of 96 compounds currently widely used. Furthermore, we also discuss the contributions of Kv1.5 and the regulation of the structure-activity relationship (SAR) of synthetic Kv1.5 inhibitors in human pathophysiology. SAR analysis is regarded as a useful strategy in structural elucidation, as it relates to the characteristics that improve compounds targeting Kv1.5. Herein, we present previous studies regarding the structural, pharmacological, and SAR information of the Kv1.5 modulator, through which we can assist in identifying and designing potent and specific Kv1.5 inhibitors in the treatment of diseases involving Kv1.5 activity.

## 1. Introduction

The voltage-gated potassium channel Kv1.5, which mediates the cardiac ultra-rapid delayed-rectifier (*I*_Kur_) current in cells [1], is an attractive familial atrial fibrillation (AF) type 7 drug target, because it is selectively expressed in the atria but not in the ventricles of human cells [2]. AF is the most common cardiac arrhythmia facing physicians, afflicting 13% of men and 11% of women over 85 years of age. In atrial tissue from AF donors, the inhibition of *I*_Kur_ extends the repolarization phase of the atrial cardiac action potential, thereby providing desirable antiarrhythmic effects without the risk of drug-induced torsade de pointes. It is noteworthy that loss-of-function Kv1.5 mutations are associated with AF, and many companies are currently exploring *I*_Kur_ modulators for the treatment of AF [3].

**Figure 1:**
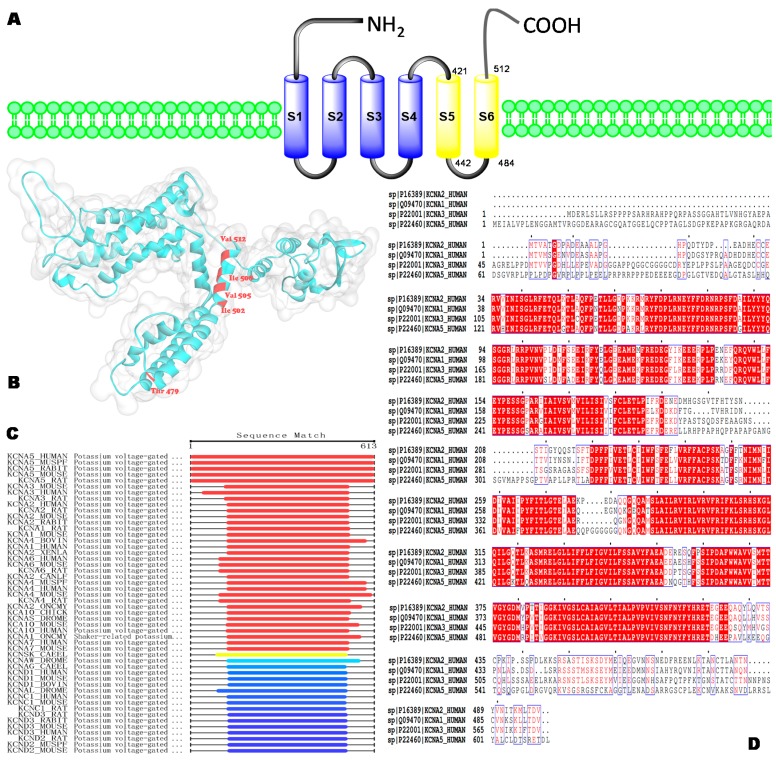
(**A**) Schematic representation of the *h*Kv1.5 *α*-subunit with the sequence of the S6 region listed. (**B**) Homologous model of Kv1.5 (Q61672) with 67.2% similarity for the Kv1.5 sequence, obtained from the SWISS-MODEL database; some of the residues are slightly different from those published in previous research. (**C**) Basic Local Alignment Search Tool (BLAST) result of KCNA5_HUMAN (P22460), obtained from the NCBI BLAST+ database. (**D**) Sequence alignment ofKCNA1_HUMAN (Q09470), KCNA3_HUMAN (P22001), KCNA2_HUMAN (P16389), and KCNA5_HUMAN (P22460), acquired from the ESPript database.

The Kv1.5 protein is encoded by the KCNA5 gene with a length of 602 amino acids in mice (Unitprot Entry: Q61762) and rat (Unitprot Entry: P19024) sequences and 613 amino acids in the human sequence (Unitprot Entry: P22460). According to the Basic Local Alignment Search Tool (BLAST) result, the sequence of Kv1.5 is similar to homology targets Kv1.1, Kv1.2, and Kv1.3 in most regions, whereas differences mainly occur toward the start and end terminals of the sequence (see Figure 1C,D). The Kv1.5 channel belongs to the shaker-type voltage-gated K^+^ channel family, and it comprises four pore-forming *α*-subunits, each containing six transmembrane segments, named S1–S6 [4,5]. A pore region is formed between the pore helix and S6 domain of each subunit, which contains the selectivity filter through which K^+^ ions flow across the plasma membrane [6,7]. Currently, the structure of the Kv1.5 protein is still awaiting identification; however, alanine-scanning mutagenesis and homologous modeling studies provide us with some amino acids, including Thr479, Ile502, Val505, Ile508, and Val512, which reside within the deep pore (Thr479-Val481) and lower S6 (Cys500-Val512) regions as putative binding sites for open-channel blockers [8,9,10,11,12,13] (Figure 1B). This not only helps us to understand the drug targets more comprehensively, but also saves time with regard to the development of potential clinical candidates in the future. From this perspective, we highlight recent advances in the discovery of small molecules as modulators of Kv1.5, and we discuss the structure-activity relationship (SAR) studies of currently used synthetic Kv1.5 inhibitors.

## 2. Summarization of Models and Mechanisms of Kv1.5 Modulators

**Figure 2:**
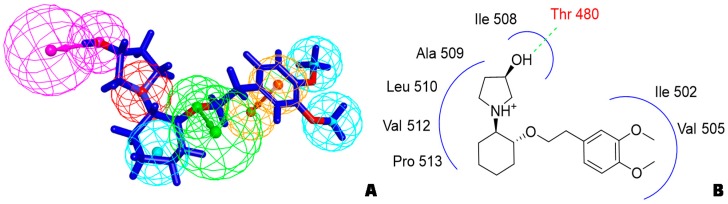
(**A**) Pharmacophore model of vernakalant (cyan ball: hydrophobic center; yellow ball: aromatic center; green ball: hydrogen bond receptor; pink ball: hydrogen bond donor; red ball: ionizable positive center); (**B**) potential binding domain of vernakalant in Kv1.5 (H-bond is expressed as green dashed).

To date, various kinds of Kv1.5 modulators have been disclosed, herein, we summarize the molecular structures and functionality of different types of Kv1.5 modulators with their chemical structure as follows (Table 1, Figure 2). As shown in Table 1, the existing Kv1.5 modulators can be divided into four categories: clinical cardiovascular drugs (**1**–**14**), other clinical drugs (**15**–**28**), drugs in development (**29**–**37**), and natural products (**38**–**56**). With the development of pharmacology, more and more experiment models including rats, HEK cells, CHO cells, Xenopus laevis oocytes, and Ltk^-^ cells have been used to evaluate the effect of Kv1.5 channel modulators; the parameters containing mRNA expression, *I*_Kur_, effective refractory period (ERP), and action potential duration (APD) were utilized to reveal the improvement degree of AF. In principle, the Kv1.5 modulators can lengthen the time course of ERP and APD to protect heart from the harm of AF.

Although the structure of Kv1.5 protein has not been characterized yet, current researches provide information for the development of Kv1.5 inhibitors according to fragment-based drug design and structure-based drug design. In regard to the design of Kv1.5 inhibitor, for the instance of the typical candidate vernakalant, in the pharmacophore model, hydrogen bond receptor, hydrogen bond donor, and hydrophobic groups should be present in the structure (Figure 2A) to play a role in the transmembrane effect to interact with the Kv1.5 channel. From the potential binding domain of vernakalant in Kv1.5 [8,14] (Figure 2B), we can see that the positively charged moiety bound in the cationophilic inner pore (mainly formed by electron-donating residues including alanine, leucine, and valine) formed a cationic “blocking particle” causing a block of the potassium channel; additionally, the uncharged dimethoxyphenyl moiety of a vernakalant has a tendency to bind in hydrophobic subunit interfaces including residues Ile 502 and Val 505. Functionally important residue isoleucine I502 in the inner helix S6 is exposed into the subunit interface of the pore module rather than into the inner pore. It is worth noting that mutations of Ile 502 decrease the potency of vernakalant, flecainide, and AVE0118, which are the ligands with a long hydrophobic tail in the side chain of the structure.

It seems that the introduction of heterocyclic rings including pyrrole (vernakalant, bepridil, clemizole, and BMS-394136) and piperdine (lobeline, CD-160130, bupivacaine, paroxetine, and donepezil) is important because these moieties usually influence the acidification conditions of the molecules, in which a potentially protonated and thus positively charged drug may enter deeply into the channel pore in a voltage-dependent way [15].

As a result of the definite curative effects and pharmacokinetic parameters proved by clinical trials, conventional drugs in new use trends seem to be a feasible way to develop new therapy. Multiple cardiovascular drugs not designed for targeting Kv1.5 have shown Kv1.5 inhibitory effect including quinidine (**9**) and diltiazem (**10**), however, the selectivity of these compounds on Kv1.5 still needs to be investigated.

As for other clinical drugs, CNS agents include: donepezil (**15**), which is generally used as an anti-Alzheimer’s agent; paroxetine (**16**), fluoxetine (**17**), and sertraline (**18**), which are usually used as antidepressant agents; and bupivacaine (**23**), propofol (**24**), midazolam (**25**), tolbutamide (**26**), and benzocaine (**27**), which are utilized as anesthetic agents. *h*ERGs (human ether-à-go-go-related genes) are widely associated with CNS diseases [16,17,18], thus it is not strange that active CNS agents can effectively modulate Kv1.5 according to the homology of the protein. Especially the neurotransmitter acetylcholine, which is an important substance that modulates the acetylcholine-activated K^+^ current [19], however, only the piperidine type acetylcholine inhibitor donepezil showed significant inhibitory effect on Kv1.5, the same phenomenon was not present in another inhibitor tacrine [15], suggesting the selectivity of the binding site of Kv1.5.

Generally, Kv1.5 drugs in development are not going smoothly. The projects listed in Table 1 have been discontinued till now. Effectiveness, toxicity, and druggability should be taken into account at this stage. Persistence of investigation in this field is necessary because the listed compound like AZD-7009 (**30**) can not only alleviate the suffering of patients from intermittent AF but also plays a role in relieving durative AF which continues to attack for more than 48 h [20]. The major voltage-gated K^+^ channels expressed in the vasculature are Kv1.2, Kv1.5, Kv2.1, and Kv7.4/7.5 [21]. Kv1.3, another Shaker-related family voltage-gated K^+^ channel, is closely related to the *h*ERG channels regulated by Kv1.1 [22], which are the important targets influencing the prolongation of Q band to the end of T band (QT) syndrome and torsade pointes attributed to the gain-of-function mutations of clinical candidates whose details are being requested by drug regulatory authorities. Limitations in the ability of high-throughput screening methods to monitor the complex behavior of *h*ERG have restricted the discovery of activators. It is noteworthy that some inhibitors of Kv1.5 channels listed in Table 1 are not specific voltage-gated K^+^ channels for Kv1.5, and some of which also block Kv1.3 channels (e.g., 4-aminopiridine (**2**), nifedipine (**6**), diltiazem (**10**), tetraethylammonium (**11**), propofol (**24**) [23], resveratrol (**52**) [24], and correolide (**55**)). Application of these drugs may result in side effects related to the inhibition of Kv1.3 channels like immune suppression, thus more attention should be paid to the toxicity to *h*ERG-related targets of Kv1.5 developing candidates. Additionally, in the field of immunization [25], nuclear factor erythroid 2-related factor (Nrf2)-induced oxidative stress-inducible protein 1/p62 enhances the inhibition of pulmonary arterial Kv1.5 channels under acute hypoxia, and the 1/p62-Kv1.3-integrin axis provides novel insight into the molecular mechanisms underlying redox-regulated cell signaling in stress-induced biological responses, which broaden future potential directions.

A variety of natural products have been proven to modulate Kv1.5, but the exploration of novel skeleton could be helpful for the current dilemma. Among the isolated compounds, the main types are terpenoids (**38**–**41**), alakaloids (**42**–**47**), and flavonoids (**48**–**50**). Terpenoids are widely reported to inhibit potassium channels [26,27,28], however, the stability and difficulty in preparation because of the lack of a fluorescence group and the abundance in chiral carbon are worth worrying about in the development. Alkaloids, as well as polypeptides like kaliotoxin (**54**) and toxins from marine animals like tetrodotoxin, have been disclosed to inhibit ion channel activity, but the toxicity of these types of compounds is also concerning; after all, *h*ERG toxicity has attracted the attention of the FDA and drugs like bepridil have been withdrawn because of their toxicity [29]. Bioactive flavonoids are also proven to modulate the Kv1.5 channel; among them is quercetin (**50**), a minor compound and activator of Kv1.5, with the tendency of developing flavonoids and phenols as health care products or food additives.This class of compounds may play a role in the daily prevention against Kv1.5 disease.

## 3. Synthetic Kv1.5 Inhibitors and SAR Investigations

In this section we collated information about chemical synthesis, pharmacological properties, and SAR investigations in the published literature from 2003 to 2019 and summarized them in a timeline. The previous work was briefly introduced in the description ofthe potential synthetic derivatives and chemical structure of compounds, and the SAR studies are listed in the corresponding figures in the perspective of medicinal chemistry. As we can see, multiple scaffolds include 5-methoxypsoralen (**60**,**68**), tetrahydroindolone (**62**–**65**), benzopyran sulfonamides (**70**–**72**), dihydropyrazolopyrimidine (**73**,**81**), and phenylquinazoline (**90**–**92**). Compounds (**86**–**88**) have been reported to be effective in inhibiting Kv1.5, suggesting potential future directions for investigations about Kv1.5 inhibitors. It is noteworthy that research from Bristol-Myers Squibb has contributed greatly with data about pharmacology and pharmacokinetics of active compounds in blocking Kv1.5, increasing the possibility that we can conquer the diseases targeting Kv1.5.

**Figure 3:**
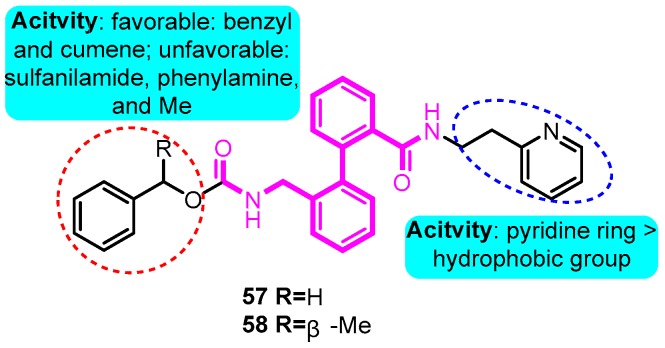
Biphenyl derivatives.

In 2003, Peukert and co-workers [80] synthesized a series of ortho-disubstituted bisaryl compounds as blockers of the Kv1.5 channel. Among the derivatives, the most potent compounds **57** (IC_50_: 0.7 μM) and **58** (IC_50_: 0.16 μM) inhibited the Kv1.5 channel with sub-micromolar half-blocking concentrations and displayed three fold selectivity over Kv1.3 and no significant effect on the *h*ERG channel and sodium currents (Figure 3).

**Figure 4:**
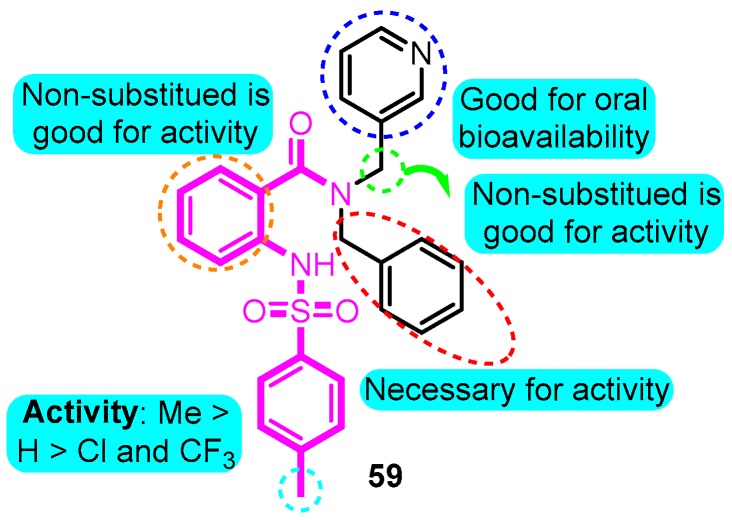
Anthranilic amides.

In 2004, Peukert et al. [81] synthesized several anthranilic amides as novel blockers of the Kv1.5 channel. The most hopeful analogue **59** showed moderate Kv1.5 inhibition (IC_50_: 0.7 μM) with good oral bioavailability, however, no significant effect on the *I*_Kr_ current of **59** was detected (Figure 4).

**Figure 5:**
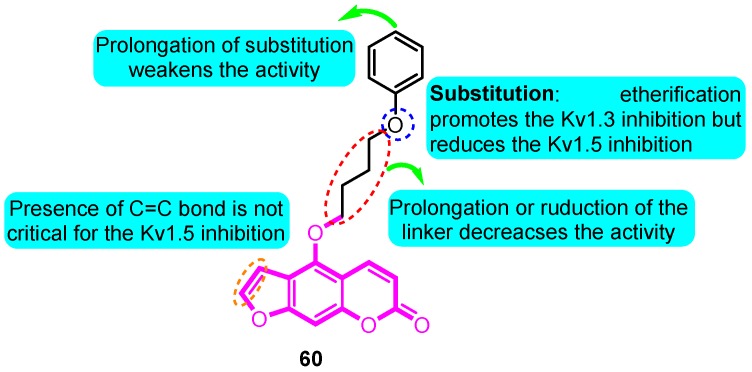
Phenoxyalkoxypsoralen analogues.

Inspired from the precursor 5-methoxypsoralen isolated from *Rutagraveolens*, Schmitz and colleagues [82] prepared a series of phenoxyalkoxypsoralen analogues and evaluated their voltage-gated ion channel blocker potency. The most potent and “druglike” compound of this series, 5-(4-phenoxybutoxy) psoralen (PAP-1, **60**), blocks Kv1.3 in a use-dependent manner, with a Hill coefficient of 2 and an EC_50_ of 2 nM, by preferentially binding to the C-type inactivated state of the channel. PAP-1 is 23 fold selective over Kv1.5, 33–125 fold selective over other Kv1 family channels, and 500–7500 fold selective over Kv2.1, Kv3.1, Kv3.2, Kv4.2, *h*ERG, calcium-activated K channels, Na, Ca, and Cl channels. PAP-1 does not exhibit cytotoxic or phototoxic effects, is negative in the Ames test, and affects cytochrome P450-dependent enzymes only at micromolar concentrations (Figure 5).

**Figure 6:**
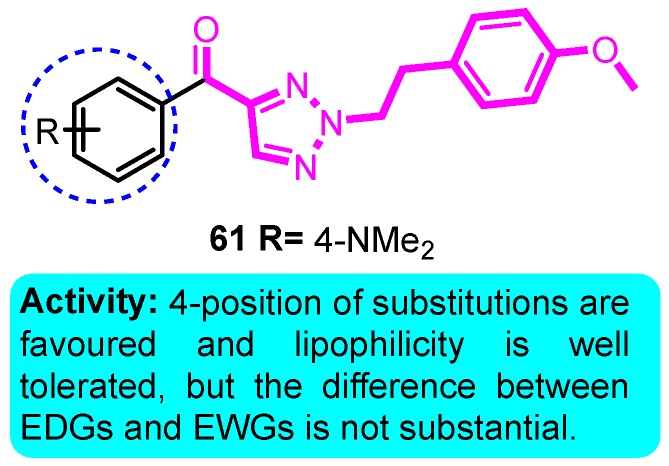
(2-phenethyl-2*H*-1,2,3-triazol-4-yl)(phenyl) methanones.

In 2006, Blass et al. [83] synthesized a cluster of (2-phenethyl-2*H*-1,2,3-triazol-4-yl) (phenyl) methanone and examined for utility as Kv1.5 channel blockers for the treatment of atrial fibrillation. The results showed that O substitution in the 4-position of the acetophenone-derived portion of the scaffold is highly favored, and the most active compound **61** blockaded Kv1.5 for 99% at a concentration of 1 μM (Figure 6).

**Figure 7:**
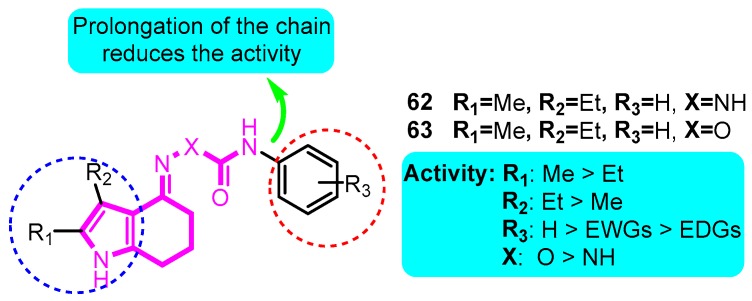
Tetrahydroindolone-derived carbamates.

Fluxe and co-workers [84] synthesized multiple tetrahydroindolone-derived carbamates as potent Kv1.5 blockers. The most promising analogues **62** and **63** exhibited the strongest Kv1.5 inhibitory effect with IC_50_ values of 67 and 21 nM, respectively. They were also very selective over *h*ERG (> 450 fold) and L-type calcium channels (> 450 fold) (Figure 7).

**Figure 8:**
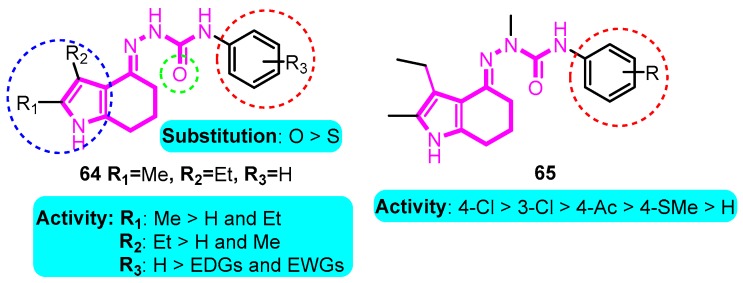
Tetrahydroindolone-derived semicarbazones.

Subsequently, Wu et al. [85] designed and synthesized tetrahydroindolone derived semicarbazones as selective Kv1.5 blockers. Compounds **64** and **65** showed good selectivity for the blockade of Kv1.5 (IC_50_: 0.13 μM for two compounds), moreover, in an anesthetized pig model, compounds **64** and **65** increased atrial ERP by about 28% and 18%, respectively, in the right atrium without affecting ventricular ERP (Figure 8).

**Figure 9:**
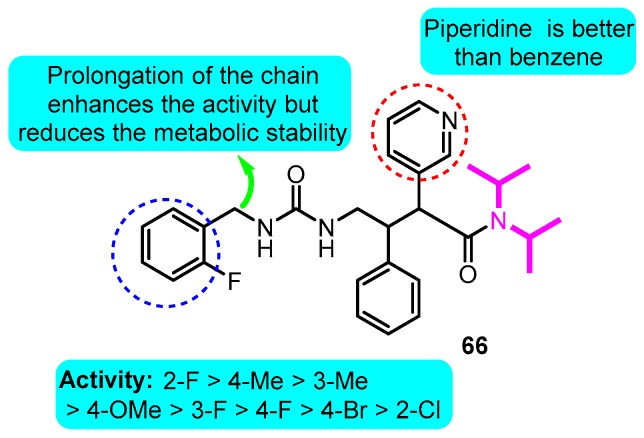
Diisopropyl amide derivatives.

Based on a diisopropyl amide scaffold, a series of potent Kv1.5 ion channel antagonists were synthesized by Nanda and colleagues [86]. The most active derivative **66**, which was a single active enantiomer of the diastereomerically pure racemic analog, exhibited significant atrial-selective effects in an in vivo model (IC_50_: 150 nM) (Figure 9).

**Figure 10:**
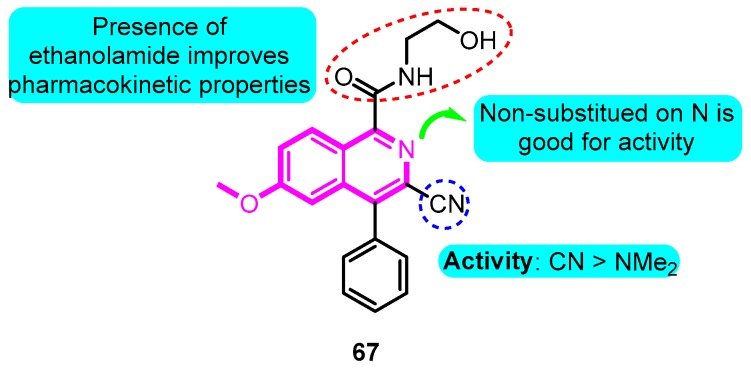
Isoquinoline-3-nitriles.

Trotter and co-workers [87] designed and synthesized a group of isoquinoline-3-nitriles as orally Kv1.5 antagonists for the treatment of AF. The ethanolamide derivative **67** exhibited improved potency (Kv1.5 HT-Clamp IC_50_: 60 nM), excellent selectivity versus *h*ERG, and good pharmacokinetic properties. Rat EP experiments confirmed that the compound potently increased ARP without significant effects on AVRP^−^ (Figure 10).

**Figure 11:**
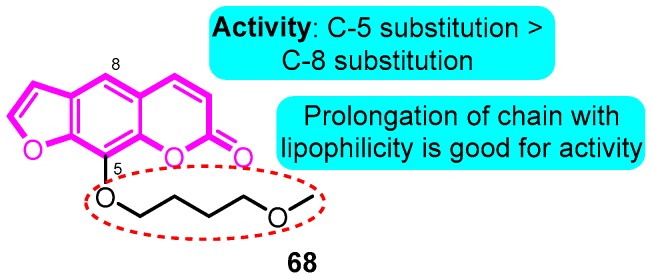
Psoralen derivatives.

In 2007, Eun et al. [88] synthesized multiple psoralen derivatives as *h*Kvl.5 channel blockers. Among them, compound **68** was the most potent in blocking *h*Kv1.5 (IC_50_: 27.4 nM), much stronger than the lead compound psoralen. Compound **68** accelerated the inactivation kinetics of the *h*Kvl.5 channel and slowed the deactivation kinetics of the *h*Kv1.5 current resulting in a tail crossover phenomenon. Compound **68** inhibited the *h*Kvl.5 current in a use-dependent manner (Figure 11).

**Figure 12:**
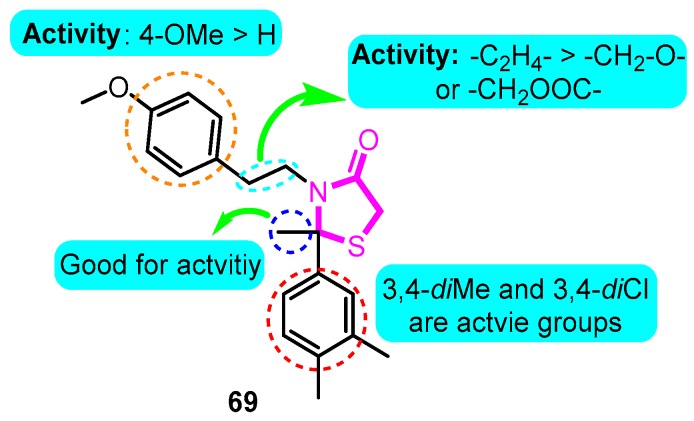
Thiazolidine derivatives.

Jackson and co-workers [89] prepared several classes of thiazolidine-based Kv1.5 blockers. The most promising analogue **69** derived from 3,4-dimethylacetophenone exhibited the strongest inhibitory effect with an IC_50_ value of 69 nM (Figure 12).

**Figure 13:**
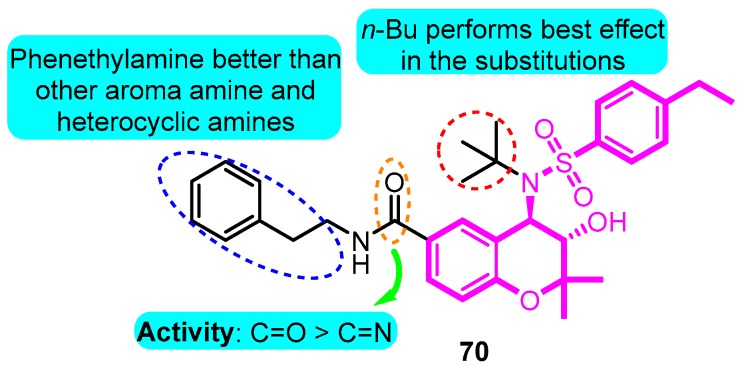
Benzopyran sulfonamides.

Lloyd et al. [90] synthesized a series of benzopyran sulfonamides and determined Kv1.5 potassium channel blocking effects. Among the productions, derivative **70** exhibited the most significant activity (IC_50_: 57 nM), and a moderate inhibition (35%) of *h*ERG at a concentration of 10 μM (Figure 13).

**Figure 14:**
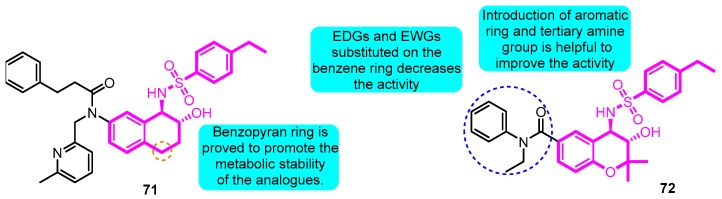
Thiazolidine derivatives.

In 2008, benzopyran sulfonamides derivatives were further investigated [91]. Compound **71** and **72** were considered as the most active derivatives in the two series of compounds with IC_50_ values of 46 and 378 nM in the inhibition of current in a L-929 cell model, respectively. Additionally, at the concentration of 1.0 μM, compound **72** displayed the most significant inbitory effect in the current of L-929 cells with an inhibitory ratio of 89% (Figure 14).

**Figure 15:**
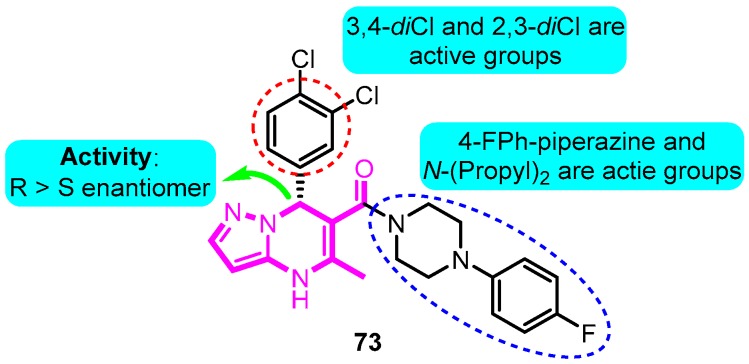
Dihydropyrazolopyrimidine derivatives.

Vaccaro and co-workers [90] synthesized a series of dihydropyrazolopyrimidine analogues as Kv1.5 inhibitors. The most promising compound **73** showed the best potential in suppressing Kv1.5, with inhibitory effects on *h*ERG (69%) and *I*_Na_^10^ (42%) at a concentration of 10 μM (Figure 15).

**Figure 16:**
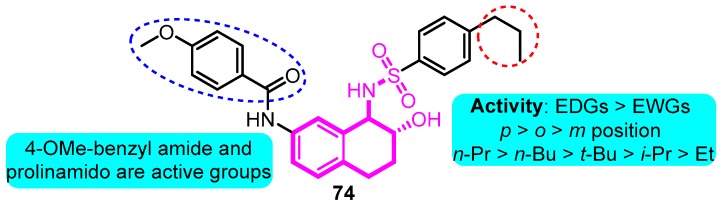
Aryl sulfonamido tetralin derivatives.

In 2008, Gross and co-workers [92] synthesized aryl sulfonamido tetralin as a Kv1.5 inhibitor according to the basis of previous work. Among the productions, compound **74** exhibited remarkable Kv1.5 inhibitions with an IC_50_ value of 90 nM; in addition, moderate *h*ERG inhibition was detected at the dose of 10 μM (39%), indicating the potential for further development of clinical candidates (Figure 16).

**Figure 17:**
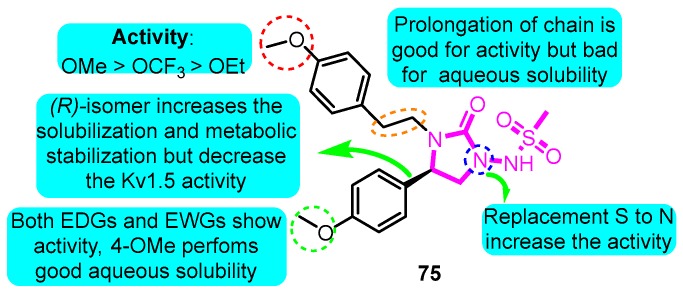
Structure-activity relationship (SAR) of imidazolidinone derivatives.

According to the structure of marketed drugs amiodarone and vernakalant, Blass et al. [93] synthesized a series of imidazolidinone derivatives as a potential treatment for atrial arrhythmia. KVI-020/WYE-160020 (**75**) exhibited the efficacy in clinically relevant models of AF and mechanistic models of the cardiac action potential with acceptable pharmacokinetic and pharmaceutical properties. The pharmacology IC_50_ values for compound **75** in Kv1.5, *h*ERG, Nav1.5, Cav1.3, Cav1.2, Kv1.1, Kv1.3, and Kv4.3 were 0.48, 15.1, >30, 23.4, >30, 2.66, 1.41, and 3.87 μM in vitro, respectively (Figure 17).

**Figure 18:**
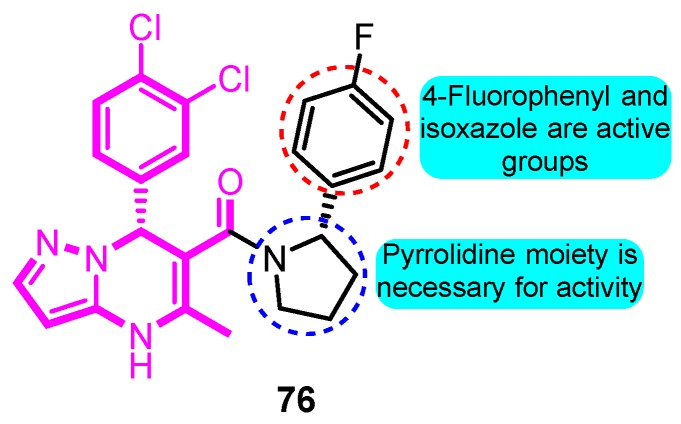
SAR of pyrazolodihydropyrimidines.

In 2010, Lloyd and co-workers [58] developed a series of pyrazolodihydropyrimidines as potent and selective Kv1.5 blockers based on previous studies. The most promising analogue BMS-394136 (**76**) displayed excellent activity in blocking Kv1.5 (IC_50_: 50 nM) and very good selectivity over *h*ERG, sodium, and L-type calcium ion channels with good pharmacokinetic parameters (Figure 18).

**Figure 19:**
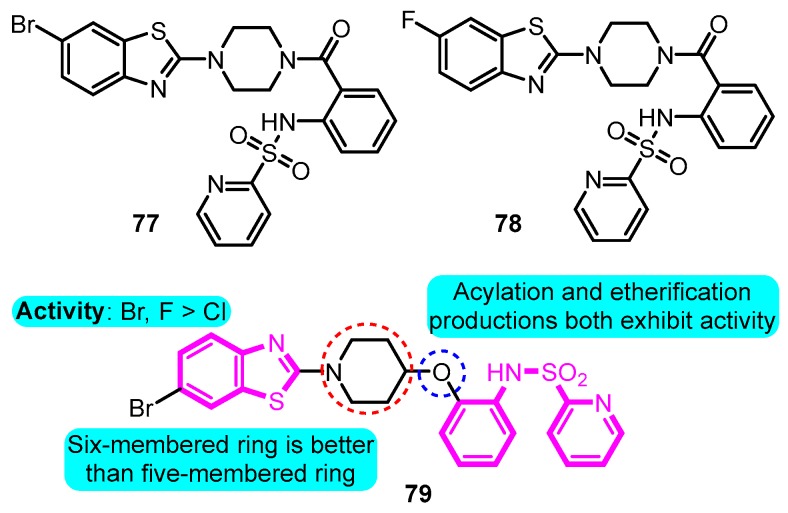
SAR of heteroarylsulfonamides.

In 2012, Blass [94] prepared several heteroarylsulfonamides as Kv1.5 inhibitors. The active analogues **77**, **78** and **79** exhibited 100% inhibition of Kv1.5 using stably transfected HEK293 cells and the FLIPR potassium ion channel assay, suggesting good potential for further investigation (Figure 19).

**Figure 20:**
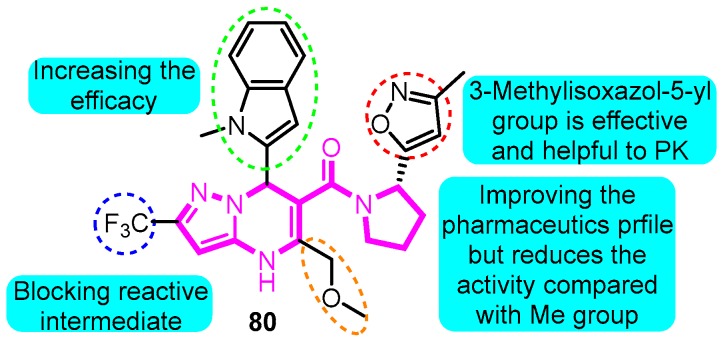
SAR of dihydropyrazolo[1,5-a]pyrimidine derivatives.

Finlay and colleagues [95] prepared several dihydropyrazolo[1,5-a]pyrimidine derivatives. Among the synthetic compounds, compound **80** showed potential to be a selective *I*_Kur_ inhibitor with Kv1.5 IC_50_ of 0.15 μM and *h*ERG with an IC_50_ value >10 μM. Furthermore, favorable pharmacokinetic properties in rats and dogs of **80** were determined; compound **80** was identified with less than 1% GSH adducts formation with an improved PK profile and equivalent PD efficacy to the lead compound (Figure 20).

**Figure 21:**
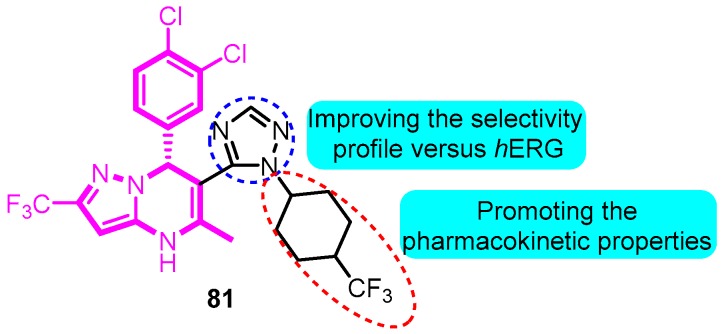
SAR of trifluoromethylcyclohexyl triazole analogues.

In 2013, triazolo and imidazo were introduced into the active scaffold dihydropyrazolopyrimidine [96]. Trifluoromethylcyclohexyl triazole analogue **81** was identified as a potent and selective Kv1.5 inhibitor (IC_50_: 133 nM) with an acceptable PK and liability profile. Compound **81** demonstrated an improved rat PK profile and was advanced to the rat PD model (Figure 21).

**Figure 22:**
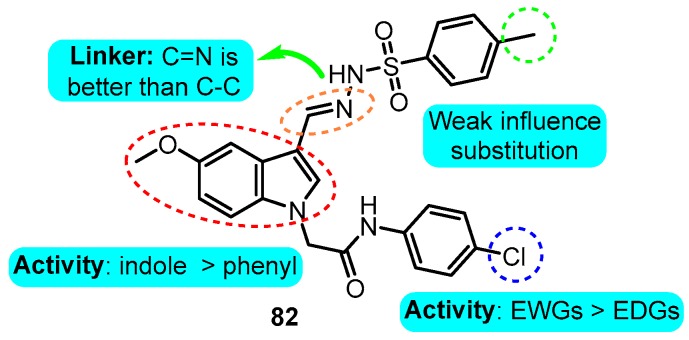
SAR of indole derivatives.

With the help of a pharmacophore model, Guo et al. [97] designed and synthesized a series of indole derivatives as potent Kv1.5 inhibitors. The most promising compound **82** displayed significant *I*_Na_, HEK 293 *h*Kv1.5, and CHO *h*ERG inhibitory activities with IC_50_ values of 52.6, 0.51, and 418.35 μM, respectively, which displayed remarkable selectivity and ameliorating effects on atrial effective refractory period (AERP) and VERP (Figure 22).

**Figure 23:**
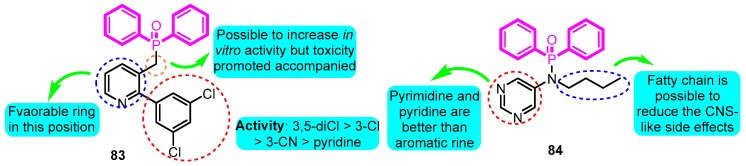
SAR of diphenylphosphinic amides and diphenylphosphine oxides.

Olsson and co-workers [98] possessed design and pharmacological evaluation of multiple potential hits targeting on Kv1.5. The compound **83** performed the best in vitro activity with Kv1.5 IC_50_ of 0.08 μM in diphenylphosphinic amide and diphenylphosphine oxide analogues (Figure 23). However, both *h*ERG and IKs active and remarkable safety in rats of compound **83** was detected and judged unsuitable for in vivo testing; conversely, the derivative **84** was regarded as a hopeful compound for further development with Kv1.5 IC_50_, IKs, C_eu20_, and QT_max_ change values for 1.0 μM, >33%, 0.6 μM, and <10%, respectively.

**Figure 24:**
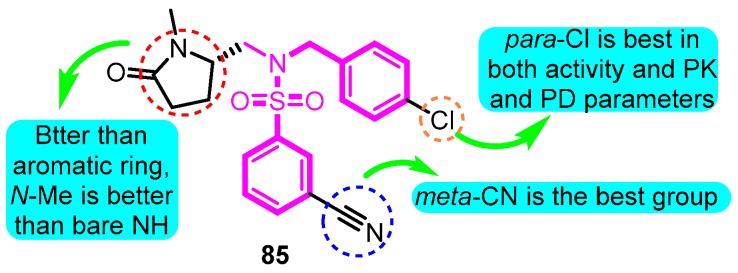
SAR of lactam sulfonamides.

In 2014, the subsequent study was updated [99], and a series of lactam sulfonamide derivatives was prepared and the Kv1.5 inhibitory potency was evaluated. The most promising candidate **85** inhibited Kv1.5 with an IC_50_ value of 0.21 μM and caused a marked increase in the atrium ERP with a C_eu20_ of 0.35 μM, which was at the same order of magnitude as the IC_50_ value from the human cellular assay. The human *h*ERG channel was blocked by compound **85** with an IC_50_ value of 30 μM, indicating a 140 fold margin of the *h*ERG and Kv1.5 in vitro values. No measurable change was noted in the QT-interval in the rabbit experiments, which also indicated a good margin to block of the *h*ERG channel. The compound **85** was well tolerated in rabbits with no signs of the CNS-like side effects observed for other Kv1.5 blockers (Figure 24).

**Figure 25:**
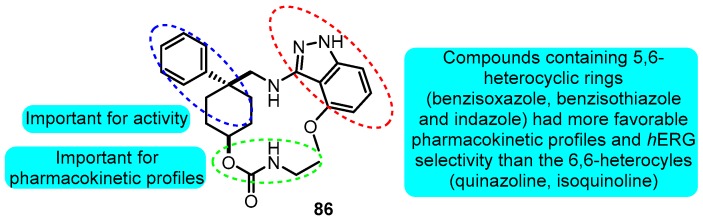
SAR of phenethylaminoheterocycles.

Johnson et al. [100] synthesized phenethylaminoheterocycles and assayed for inhibition of the Kv1.5 potassium ion channel as a potential approach to the treatment of atrial fibrillation. Combination of the indazole with a cyclohexane-based template gave the most promising derivative **86** (Kv1.5 IC_50_: 138 nM) which demonstrated significant prolongation of AERP in the rabbit pharmacodynamic model (Figure 25).

**Figure 26:**
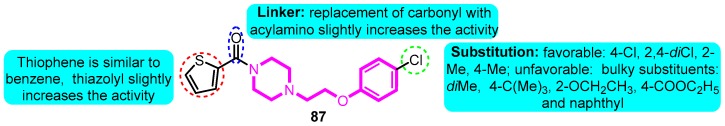
SAR of 1-aryloxyethyl piperazine derivatives.

Guo and colleagues [101] prepared a series of 1-aryloxyethyl piperazine derivatives as Kv1.5 potassium channel inhibitors. The most potent compound **87** exerted significant activity on *h*Kv1.5 (IC_50_: 0.72 μM), balanced Log D, and permeability. In addition, comparable in vivo potency with sotalol and dronedarone and remarkable safety in rats of compound **87** were detected as well (Figure 26).

**Figure 27:**
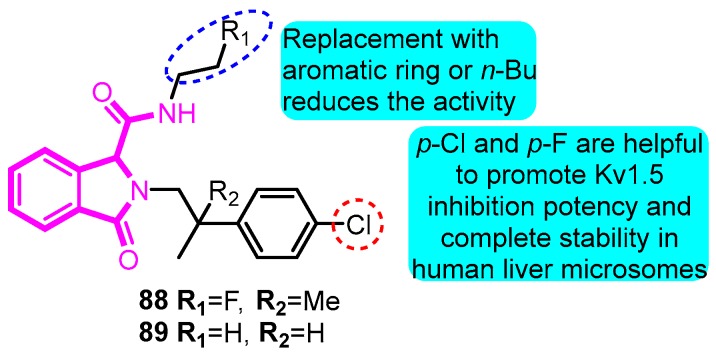
SAR of isoindolinones.

In 2016, Kajanus et al. [102] synthesized multiple isoindolinone compounds as Kv1.5 blockers. The most potent compounds **88** and **89** exhibited an inhibitory effect with the IC_50_ values of 0.4 and 0.7 µM on Kv1.5, respectively. The above-mentioned two compounds were found to have desirable in vivo PK properties in a mouse model (Figure 27).

**Figure 28:**
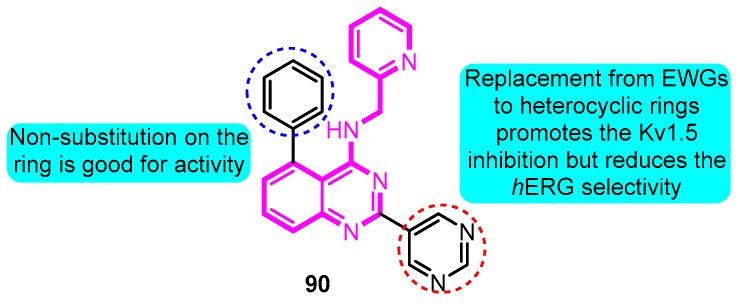
SAR of phenylquinazoline derivatives.

Finlay and co-workers [103] explored phenylquinazoline derivatives as Kv1.5 inhibitors. 5-Phenyl-*N*-(pyridin-2-ylmethyl)-2-(pyrimidin-5-yl)quinazolin-4-amine (**90**) was identified as a potent and ion channel selective inhibitor (Kv1.5 IC_50_: 90 nM, *h*ERG inhibition: 43% at 10 μM) with robust efficacy in the pre-clinical rat ventricular effective refractory period (VERP) model and the rabbit atrial effective refractory period (AERP) model (Figure 28).

**Figure 29:**
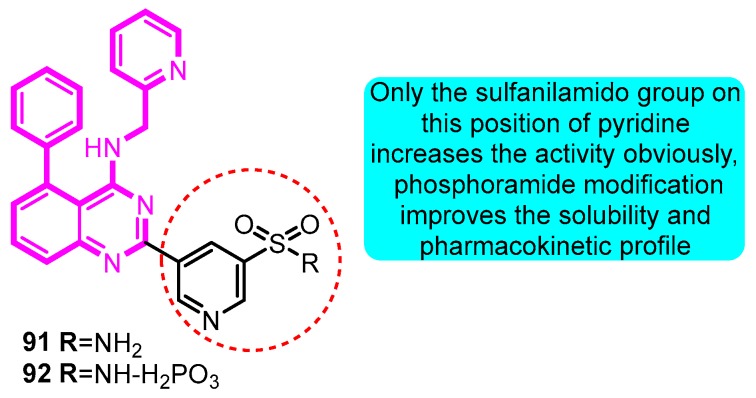
SAR of phenylquinazoline sulfonamide derivatives.

Subsequently in 2017, Gunaga et al. [58] modified the structure of **91** with a series of analogues and evaluated the *I*_Kur_ inhibitory effect. 5-[5-Phenyl-4-(pyridin-2-ylmethylamino)-quinazolin-2-yl] pyridine-3-sulfonamide (**92**) was identified as the lead compound in this series with good selectivity over *h*ERG (Kv1.5 IC_50_: 50 nM, *h*ERG IC_50_: 1.9 μM). Compound **91** exhibited robust effects in rabbit and canine pharmacodynamic models and an acceptable cross-species pharmacokinetic profile which was then advanced as a clinical candidate. Further optimization of **91** to mitigate pH-dependent absorption resulted in identification of the corresponding phosphoramide prodrug (**92**) with an improved solubility and pharmacokinetic profile (Figure 29).

**Figure 30:**
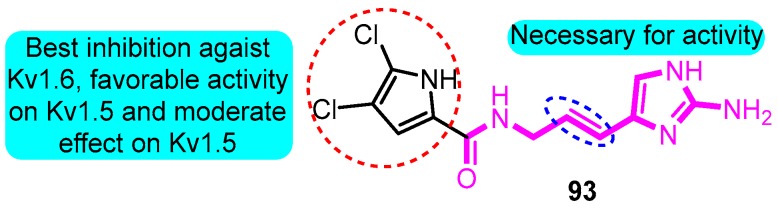
SAR of oroidin derivatives.

According to the skeleton of *Agelas* alkaloids clathrodin, oroidin, and hymenidin, Zidar and colleagues [104] synthesized multiple derivatives as inhibitors of the voltage-gated potassium channels. The most potent inhibitor was (*E*)-*N*-(3-(2-amino-1H-imidazol-4-yl)allyl)-4,5-dichloro-1H-pyrrole-2-carboxamide (**93**) with IC_50_ values between 1.4 and 6.1 mM against Kv1.3, Kv1.4, Kv1.5, and Kv1.6 channels (Kv1.5 IC_50_: 6.1 μM) (Figure 30).

**Figure 31:**
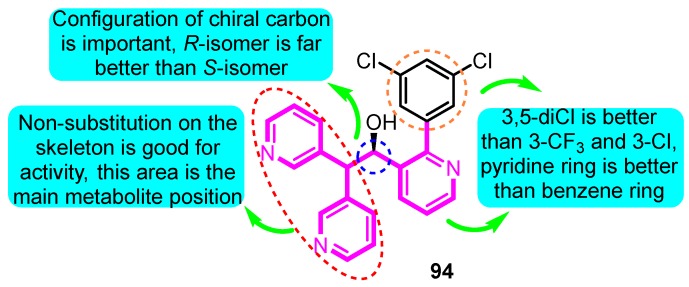
SAR of oroidin MK-1832.

Wolkenberg et al. [105] told the story of the development of prospective candidate MK-1832 (**94**) (Figure 31). Based on the structure of MK-0448, a cluster of derivatives were synthesized and tested the Kv1.5 inhibitory effect and in vivo and in vitro toxicity. MK-1832 (**94**) was considered to be the best derivative with pharmacological parameters including Kv1.5, I_kur_, and I_kr_(*h*ERG) IC_50_ values for 29, 11 and 1.28 × 10 ^5^ nM, respectively, and pharmacokinetic parameters including dog in vivo atrial refractory period EC_10_ for 14 nM and threshold change in ventricular refractory period >25 μM.

**Figure 32:**
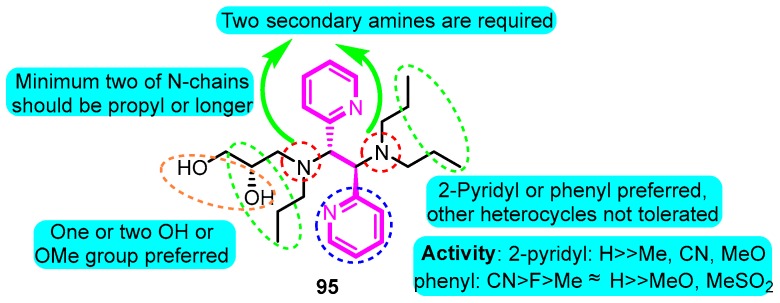
SAR of 1,2-bis(aryl)ethane-1,2-diamines.

In 2019, Kajanus and colleagues [106] prepared potassium channel blocking 1,2-bis(aryl)ethane-1,2-diamines active as antiarrhythmic agents. The most promising analogue **95** displayed significant nanomolar potency in blocking Kv1.5 in human atrial myocytes (IC_50_: 1.7 μM, *I*_Kur_ IC_50_: 60 nM) and based on the PD data, the estimated dose for men was 700 mg/day (Figure 32).

**Figure 33:**
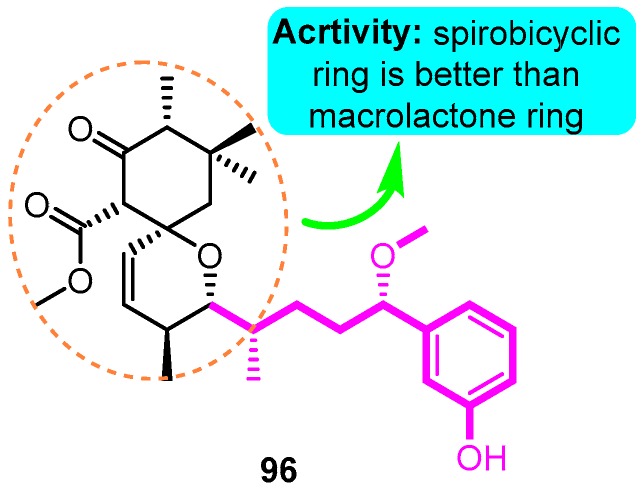
SAR of aplysiatoxin derivatives.

Recently, natural products with novel structural motif as a Kv1.5 inhibitor also gained progress in this field. In the sequence of the isolation of compound debromoaplysiatoxin A (**38**) and debromoaplysiatoxin B (**39**) [63], Tang and co-workers [14] identified other novel aplysiatoxin derivatives from the marine cyanobacterium *Lyngbya* sp. Among them, compound oscillatoxin E (**96**) with the hexane-tetrahydropyran of a spirobicyclic system skeleton exhibited the strongest Kv1.5 inhibition (IC_50_: 0.79 μM) in the CHO cells at an HP of -80 mV (Figure 33).

## 4. Conclusions

Herein the target and the pharmacological properties with structural, pharmacological, and SAR information of Kv1.5 modulators were discussed. Detailed descriptions of pharmacology parameters and SAR studies provide an actionable path forward for medicinal chemists to optimize the structure of Kv1.5 modulators. Further experiments should improve the PK and safety after the effectiveness is proven. Design and development of potential and selective Kv1.5 modulators are important and challenging tasks. Based on the existing pharmacophoric requirements and potential protein structure parsed in the future, some novel effective Kv1.5 modulators may be designed and prepared [107,108]. However, gaps exist in the scientific studies on Kv1.5 modulators. Firstly, the selectivity of existing Kv1.5 modulators remains to be investigated, and more specific modulators aiming at the Kv1.5 channel are needed in the future. Secondly, from the point of application, the market of AF is relatively small, and the sales condition of marked anti-AF agents is not satisfactory as a whole, thus more in-depth pharmacological investigation of roles of Kv1.5 are required in the future. Moreover, the definite structure of Kv1.5 protein is still vacant, difficulties and potential fallacy are still consistent in the design of modulators only estimating by the pocket of homologous models.

SAR investigation is crucial for the development of novel promising clinical candidates. It is anticipated that the information compiled in this review article not only updates researchers with the recently reported pharmacology and SAR of Kv1.5 modulators, but also motivates them to design and synthesize promising Kv1.5 modulators with improved medicinal properties.

## Figures and Tables

**Table 1:** Active Kv1.5 modulators.

No.	Name	CAS	Status	Model	Mechanism	Ref.
**Clinical Cardiovascular Drugs**						
**1**	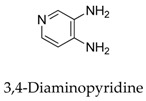	54-96-6	Approved	Smooth muscle cells	Blocking *h*Kv1.5 current with a threshold fur activation near –45 mV.	[30]
**2**	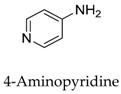	504-24-5	Approved	HEK cells	Inhibiting *h*Kv1.5 current after long-term treatment, abbreviating the prolongation of action potential duration in chronic atrial fibrillation (AF).	[31]
**3**	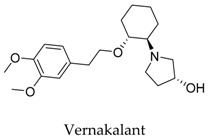	794466-70-9	Approved, investigational	HEK cells	Selective blocking of the Kv1.5 channel by interacting with important residues including Thr 479, Thr 480, Ile 502, Val 505, and Val 508.	[32]
**4**	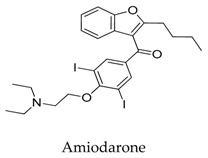	1951-25-3	Approved, investigational	Papillary muscles or single ventricular cells	Decreasing the amount of mRNA for Kv1.5.	[33]
**5**	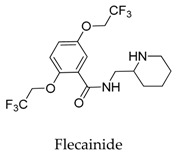	54143-55-4	Approved, withdrawn	Xenopus laevis oocytes	Producing open-channel block of Kv1.5 by sensitively interacting with key residues including Asp 469, Val 481, and Ile 502 in the S6 region of Kv1.5.	[34]
**6**	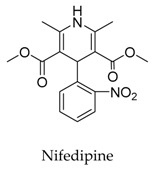	21829-25-4	Approved	HEK cells	Blocking *h*Kv1.5 channels with 6.3 μM of K_d_ was affected by mutations like Arg 487 similar to those known to affect outer pore C-type inactivation.	[35]
**7**	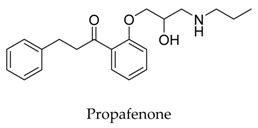	54063-53-5	Approved	Ltk^-^ cells	Inhibiting *h*Kv1.5 current with K_d_value of 9.2 μM, showing time-dependent and dose-dependent manners simultaneously.	[36]
**8**	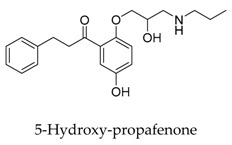	86384-10-3	-	Ltk^-^ cells	Inhibiting *h*Kv1.5 current with K_d_value of 4.4 μM, showing time-dependent and dose-dependent manners simultaneously.	[36]
**9**	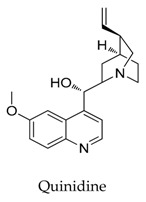	56-54-2	Approved, investigational	HEK cells	Producing a voltage-dependent block between +30 and +120 mV (K_d_ at +60 mV = 7.2 μM) with an equivalent electrical distance in the steady state.	[37]
**10**	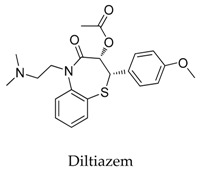	42399-41-7	Approved, investigational	CHO cells	Blocking *h*Kv1.5 channels, in a frequency-dependent manner exhibiting a biphasic dose-response curve (IC_50_: 4.8 nM and 42.3 μM) by binding to the open and inactivated state of the channels.	[38]
**11**	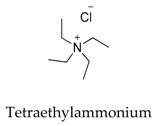	66-40-0	Experimental, investigational	BT-474 breast cancer cell	Blocking *h*Kv1.5 channels in a delayed rectifier manner.	[39]
**12**	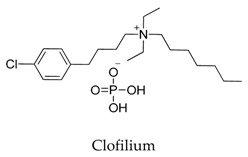	68379-03-3	-	CHO cells	Inhibiting *h*Kv1.5 current with concentration-dependent acceleration of the apparent channel inactivation in both outside-out and inside-out patches.	[40]
**13**	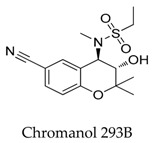	163163-23-3	-	CHO cells	Blocking *h*Kv1.5 current stereoselectivity, the results showed that (-)-[*3R, 4S*] was more potent than the (-)-enantiomer.	[41]
**14**	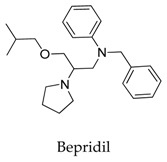	64706-54-3	Approved, withdrawn	HEK cells	Inhibiting the *h*Kv1.5 channel current with IC_50_ value of 6.6 μM.	[42]
**Other Clinical Drugs**						
**15**	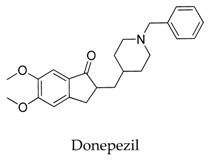	120014-06-4	Approved	HEK cells	Resulting in a rapid and reversible block of Kv1.5 currents (IC_50_: 72.5 μM) with a significant delay in the duration of activation and deactivation, and the outer mouth region proved to be the target site.	[15]
**16**	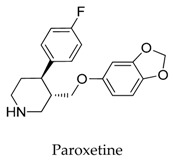	61869-08-7	Approved, investigational	CHO cells	Slowing the deactivation time course, resulting in a tail crossover phenomenon when the tail currents, recorded in the presence and absence of paroxetine, were superimposed.	[43]
**17**	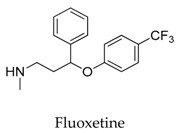	54910-89-3	Approved, vet approved	Human Pulmonary Artery Smooth Muscle Cells	Protecting against big endothelin-1 induced anti-apoptosis and rescued Kv1.5 channels in human pulmonary arterial smooth muscle cells.	[44]
**18**	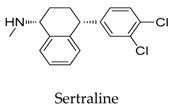	79617-96-2	Approved	CHO cells	Reducing Kv1.5 whole-cell currents in a reversible dose-dependent manner and accelerating the decay rate of inactivation of Kv1.5 currents without modifying the kinetics of current activation.	[45]
**19**	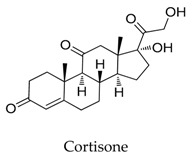	53-06-5	Approved	Xenopus oocytes	Suppressing the amplitude of Kv1.5 channel current with IC_50_ value of 50.2 μM.	[46]
**20**	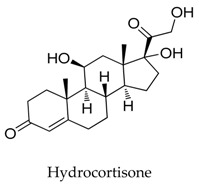	50-23-7	Approved, vet approved	Xenopus oocytes	Suppressing the amplitude of Kv1.5 channel current with IC_50_ value of 33.4 μM.	[46]
**21**	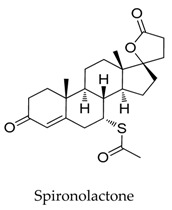	52-01-7	Approved	Male Wistar rats	Shorting the APD_90_(action potential duration) and increasing the expression of Kv1.5.	[47]
**22**	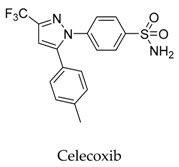	169590-42-5	Approved, investigational	Ltk^-^ cells	Blocking *h*Kv1.5 channels with an IC_50_ of 26.2 μM for the peak current and 5.5 μM for the current at the end of a 250 ms pulse to +60 mV.	[48]
**23**	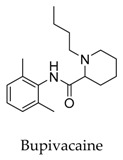	38396-39-3	Approved, investigational	Ltk^-^ cells	Blocking the opening of *h*Kv1.5 channels stereoselectivity; the results showed the K_d_ value for *R*(+)-enantiomer (4.1 μM) was six-fold more potent than the *S*(-)-enantiomer (27.3 μM).	[49,50]
**24**	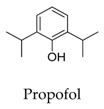	2078-54-8	Approved, investigational, vet approved	CHO cells	Inducing a time-dependent decline of the *h*Kv1.5 current (IC_50_: 62.9 μM) during depolarizing steps and slowing the time course of tail current decay upon repolarization.	[4]
**25**	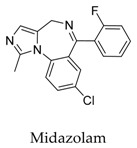	59467-70-8	Approved	HEK cells	Inhibited Kv1.5 current (IC_50_: 17 μM) without influence on the half-maximal activation voltage of Kv1.5 channels.	[51]
**26**	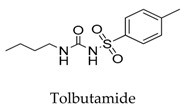	64-77-7	Approved, investigational	Insulin-secreting (INS-1) cells	Activating Kv1.5 channel and the activation of secretion can be counteracted by an excessive stimulation of Kv channels in INS-1 cells which shorten the Ca^2+^ signal and confine the insulin secretion.	[52]
**27**	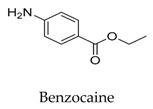	94-09-7	Approved	Ltk^-^ cells	Blocking *h*Kv1.5 channels in a voltage-dependent manner and modifying the voltage-dependence of channel activation.	[53]
**Drugs in Development**						
**28**	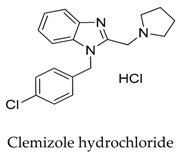	1163-36-6	Phase 2 Clinical	HEK cells	Decreasing *I*_Ks_ and human Kv1.5 channel current at doses of 3 and 10 μM at voltages ranging from –14.3 to +34.7 mV.	[54]
**29**	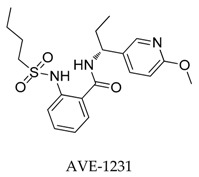	767334-89-4	Phase 1 discontinued	CHO cells	Inhibiting *h*Kv1.5 current with IC_50_ value of 3.6 μM, blocking early atrial K^+^ channels, and prolonging atrial refractoriness with no effects on electrocardiography intervals and ventricular repolarization.	[55]
**30**	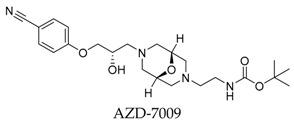	864368-79-6	Phase 2 discontinued	CHO cells	Blocking *h*Kv1.5 current with IC_50_ value of 27 μM with a slight decrease at higher frequency.	[56]
**31**	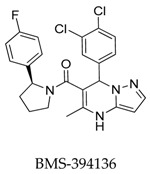	343246-73-1	Phase 1 discontinued	Mouse fibroblast L929 cells	Showing excellent activity in blocking Kv1.5 (IC_50_: 0.05 μM) and very good selectivity over *h*ERG, sodium, and L-type calcium ion channels.	[57]
**32**	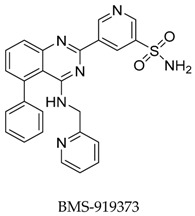	1272353-82-8	Phase 1 discontinued	Mammalian L-929 cells	Blocking *h*Kv1.5 current with IC_50_ value of 0.05 μM with an acceptable in vitroselectivity and liability profile and a good pharmacokinetic profile across species.	[58]
**33**	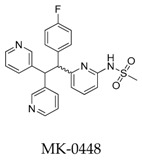	875562-81-5	Phase 1 discontinued	HK2BN9 cells	Blocking Kv1.5 current in an expression system and concentration-dependently elevated the plateau phase of atrial action potentials (APs).	[59]
**34**	XEN-D0103 (Undisclosed structure)	1410180-16-3	Phase 2 discontinued	CHO cells	Prolongating action potential duration (APD) and suppressed APs at high stimulation rates in sinus rhythm (SR) and paroxysmal AF (*p*AF) tissue.	[60]
**35**	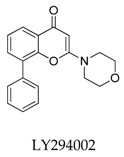	154447-36-6	Experimental	CHO cells	Acting directly on *h*Kv1.5 currents as an open channel blocker with key interacting residues located in the pore region (Thr 480, Arg 487) and the S6 segment (Ile 502, Ile 508, Leu 510, Val 516).	[9]
**36**	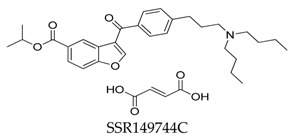	752253-75-1	-	CHO cells	Inhibiting several potassium currents including *I*_Kr_, *I*_Ks_, *I*_K(ACh)_, and *I*_Kv1.5_ at doses of 0.01–30 μM.	[61]
**37**	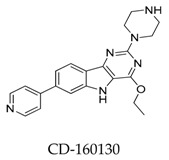	1034194-07-4	-	HEK cells	Inhibiting *h*Kv1.5 current slightly when specially blocked by the Kv11.1 channel.	[62]
***Natural Products***			**Type**			
**38**	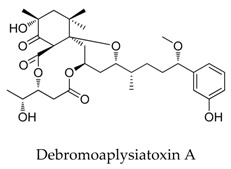	2334247-91-3	Terpenoid	CHO cells	Blocking Kv1.5 with an IC_50_ value of 6.94 μM.	[63]
**39**	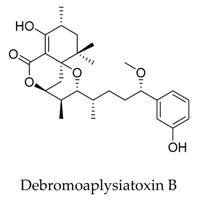	2334247-94-6	Terpenoid	CHO cells	Blocking Kv1.5 with an IC_50_ value of 0.30 μM.	[63]
**40**	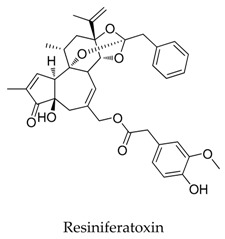	57444-62-9	Terpenoid	C6 glioma cells	Inhibiting the *h*Kv1.5 current in time and dose-dependent manners.	[64]
**41**	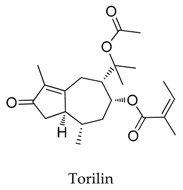	13018-10-5	Terpenoid	Ltk^-^ cells	Inhibiting the *h*Kv1.5 current in time- and voltage-dependent manners, with an IC_50_ value of 2.51 μM at +60 mV accelerated the inactivation kinetics of the *h*Kv1.5 channel and slowed the deactivation kinetics of the *h*Kv1.5 current, resulting in a tail crossover phenomenon.	[65]
**42**	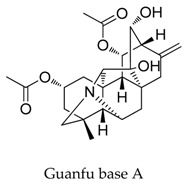	1394-48-5	Alkaloid	Guinea pigs	Blocking *I*-Kv1.5 slightly with a ratio of 20.6% at a dosage of 200 μM.	[66]
**43**	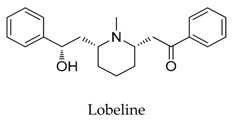	90-69-7	Alkaloid	HEK cells	Accelerating the decay rate of Kv1.5 inactivation, decreased the current amplitude at the end of the pulse in a concentration-dependent manner with an IC_50_ value of 15.1 μM.	[67]
**44**	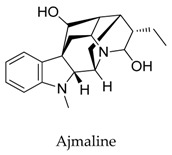	4360-12-7	Alkaloid	*Xenopus oocytes*	Inhibiting Kv1.5 with an IC_50_ of 1.70 μM in Xenopus expression system, resulting in a mild leftward shift of Kv1.5 activation curve.	[68]
**45**	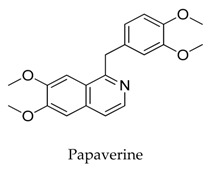	58-74-2	Alkaloid	Ltk^-^ cells	Blocking *h*Kv1.5 channels and native *h*Kv1.5 channels in a concentration-, voltage-, state-, and time-dependent manner.	[69]
**46**	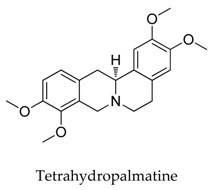	2934-97-6	Alkaloid	HEK cells	Blocking Kv1.5 currents dose-dependently with an IC_50_ value of 53.2 μM inhibited the delayed rectifier effect of Kv1.5 resulting in a potential left shift of the inactivation curve.	[70]
**47**	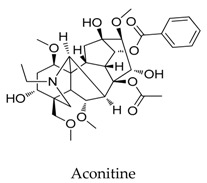	302-27-2	Alkaloid	*Xenopus laevis oocytes*	Producing a voltage-, time-, and frequency-dependent inhibition of Kv1.5 (IC_50_: 0.796 μM).	[71]
**48**	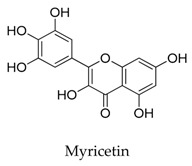	529-44-2	Flavonoid	HEK cells	Inhibiting *I*_kur_ and the expression of *h*Kv1.5 in a dose-, time-, and frequency-dependent manner.	[72]
**49**	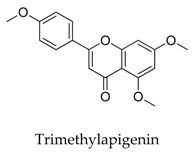	5631-70-9	Flavonoid	HEK cells	Suppressing *h*Kv1.5 current in HEK 293 cell line (IC_50_: 6.4 μM) and the ultra-rapid delayed rectify K^+^ current *I*_Kur_ in human atrial myocytes (IC_50_: 8.0 μM) by binding to open channels in a use- and frequency-dependent manner.	[73]
**50**	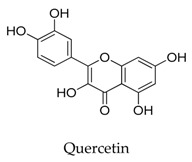	117-39-5	Flavonoid	*Xenopus laevisoocytes*	Activating *h*Kv1.5 channels (EC_50_: 37.8 μM) by interacting with key residue Ile 502 in S6 region.	[74]
**51**	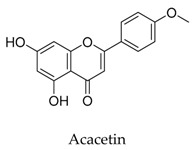	480-44-4	Flavonoid	HEK cells	Blocking open *h*Kv1.5 channels by binding to their S6 domain influenced by the interaction of V505A, I508A, and V512A.	[75]
**52**	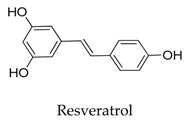	501-36-0	Phenol	Human PASMCs	Reducing the expression of Kv1.5 mRNA to reverse monocrotaline-induced pulmonary vascular and cardiac dysfunction.	[76]
**53**	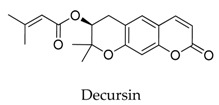	5928-25-6	Coumarin	Ltk^−^ cells	Inhibiting *h*Kv1.5 current in a concentration- and use-dependent manner, with an IC_50_ value of 2.7 μM at +60 mV accelerated the inactivation kinetics of the *h*Kv1.5 channel, resulting in a tail crossover phenomenon.	[77]
**54**	Kaliotoxin	145199-73-1	Polypeptide	T cell	Inhibiting *h*Kv1.5 current in a dose-dependent manner.	[64]
**55**	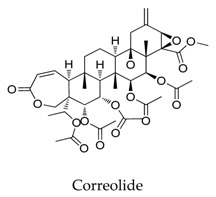	190017-00-6	Nor-triterpenoid	CHO cells	Inhibiting Kv1.5 with an IC_50_ of 1.77 μM and influenced by the mutations T480A, V505A, I508A, as well as V516A.	[78]
**56**	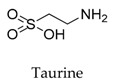	107-35-7	Amino acid	Male Wistar rats	Down-regulating the mRNA expression level of Kv1.5.	[79]

## Abbreviations

AF	Atrial fibrillation
BLAST	Basic Local Alignment Search Tool
C_eu20_	Unbound steady-state plasma concentration
CHO cells	Chinese hamster ovary cells
CNS	Central nervous system
EDGs	Electron donating groups
EWGs	Electron withdrawing groups
HEK cells	Human embryonic kidney 293 cells
*h*ERG	Human ether-à-go-go-related gene
*h*Kv1.5 channels	Human Kv1.5 channels
Human PASMCs	Human pulmonary arterial smooth muscle cells
*I*_Kur_	Cardiac ultra-rapid delayed-rectifier
IC_50_	50% inhibitory concentration
Ile	Isoleucine
Nrf2	Nuclear factor erythroid 2-related factor
SAR	Structure–activity relationship
Thr	Threonine
Val	Valine
VERP	Ventricular effective refractory period
